# Synthesis of FeOOH/Al_2_O_3_ Composites with Excellent Adsorption Performance and Regenerability for Phosphate Removal from Wastewater

**DOI:** 10.3390/molecules30214200

**Published:** 2025-10-27

**Authors:** Boning Jiang, Shuaiqi Chen, Haoran Wang, Jingwen Yan, Xuhui Wang, Xiangyu Xu, Jiaqing Song

**Affiliations:** 1 College of Chemistry, Beijing University of Chemical Technology, Beijing 100029, China; 2023400285@mail.buct.edu.cn (B.J.); 2022430033@mail.buct.edu.cn (S.C.); 15684736562@163.com (H.W.); 2023210793@mail.buct.edu.cn (J.Y.); xuxy@mail.buct.edu.cn (X.X.); 2 State Key Laboratory of Chemical Resource Engineering, Beijing University of Chemical Technology, Beijing 100029, China; 3 Sinopec Catalyst Company Limited, Beijing 100176, China; xuhuiwang@126.com

**Keywords:** alumina, iron oxyhydroxide, composite materials, phosphate removal, regeneration

## Abstract

To address the issues of insufficient capacity and difficult regeneration of adsorbents for phosphate removal from wastewater, in this study, FeOOH/Al_2_O_3_ adsorbents were successfully developed by in situ growing amorphous iron oxyhydroxide (FeOOH) within the pores of alumina (Al_2_O_3_) using a simple method. The physicochemical properties of FeOOH/Al_2_O_3_ adsorbents were characterized using X-ray Diffraction (XRD), N_2_ adsorption/desorption analysis, and scanning electron microscopy (SEM). Additionally, their phosphate adsorption properties were comparatively investigated. The results revealed that FO-A-3, one of the FeOOH/Al_2_O_3_ samples prepared with Fe/Al molar ratio of 0.47, exhibited excellent adsorption capacity and a relatively fast adsorption rate, surpassing those of Al_2_O_3_ and amorphous FeOOH alone. The adsorption process of phosphate using FO-A-3 conformed to the pseudo-second-order kinetic model and the Langmuir isotherm model, with a maximum adsorption capacity of 131.00 mg/g. To tackle the problem of poor regeneration performance, this study innovatively proposed a repeatable and simple regeneration strategy. Experiments demonstrated that FO-A-3 maintained a relatively high adsorption capacity after four cycles of regeneration.

## 1. Introduction

Despite its role as an essential component of the food chain and agricultural economy, phosphate is the culprit of water eutrophication, threatening aquatic ecosystems and human health [1]. Therefore, increasing attention is paid to the removal and recovery of phosphate from wastewater [2]. In the past few decades, extensive research has been conducted on the removal of phosphate from wastewater, such as ion exchange [3,4], chemical precipitation [5,6], biological removal processes [7,8], and adsorption [9,10]. In contrast, adsorption has been widely investigated due to its low cost, prominent efficiency, operational simplicity, and ease of recovery of phosphate [11].

Alumina (Al_2_O_3_) is commonly used as an adsorbent due to its high specific surface area, abundant pore structure, and low cost. However, Al_2_O_3_ exhibits a relatively long equilibrium adsorption time, low adsorption capacity and poor regeneration performance when used as an adsorbent for phosphate removal [12,13]. This not only increases long-term costs but also poses potential environmental risks. Therefore, it is highly necessary to further optimize Al_2_O_3_ to enhance its phosphate adsorption and regeneration performance. Jiang et al. [14] loaded molecular Co(Ni)-bpy complexes onto Ag foil, and such interactions led to the ability of active sites to adsorb on the surface to participate in the reaction. It has been revealed that composite materials modified with metal oxides not only retain the original advantages of the host material when functioning as an adsorbent or carrier, but also integrate the excellent adsorption performance of metal oxides for phosphate [15,16]. Wang et al. [17] prepared FeOOH/γ-Al_2_O_3_ (AOF) using the sol–gel method and conducted treatment of arsenic-contaminated groundwater. The results demonstrated that FeOOH (GOE) enhanced the adsorption capacity of Al_2_O_3_ (AO) because arsenic species were simultaneously adsorbed on the surfaces of both GOE and AO. These arsenic removal studies offer valuable references for the application of FeOOH and Al_2_O_3_ composites in phosphate removal, and also provide a feasible solution for preparing a composite material with excellent phosphate adsorption performance.

Iron oxyhydroxide (FeOOH) is commonly used as an adsorbent for phosphate removal due to its abundant reserves and sufficient affinity for phosphate [18,19,20]. The reactivity has been proven to be highly size-dependent [21,22]. Zhang et al. [23] prepared amorphous Fe-P alloys, and they showed superior HER and OER performance partly because of their fast interfacial charge transfer ability resulting from the amorphous structure. Zhang et al. [24] have also confirmed that amorphous FeOOH (AF) contains more reactive adsorption sites (μ_3_-OH) than well-crystallized FeOOH (CF). However, industrial production still poses challenges as smaller particles are more prone to aggregation [25,26,27], which can be resolved by immobilizing amorphous FeOOH on a suitable support [28,29]. In this way, more reactive adsorption sites can be exposed. Ai et al. [26] developed a FeOOH/BF composite by immobilizing FeOOH on basalt fiber (BF), and the maximum adsorption capacity of phosphate was significantly increased to 39.08 mg/g. Tao et al. [30] prepared FeOOH@MS composites using melamine sponge (MS) as a support. The adsorbent with a mass ratio of 5:1 for Fe to MS showed a higher capacity (115.5 mg/g).

Currently, elution stands as the most commonly employed method for regeneration of metal-based adsorbents, with common leaching solutions including acids, alkalis, and salt solutions [31]. Shi et al. [32] prepared a nanocomposite adsorbent MALZ by anchoring nanostructured ternary (hydr)oxides of Mg–Al–La onto zeolite, and this adsorbent was regenerated using a 0.5 M NaOH solution. However, after four cycles, it showed a 24.2% decrease in adsorption capacity compared to the pristine MALZ. One of the speculated reasons for this phenomenon is the leaching of some active sites (ternary (hydr)oxides). Therefore, if adsorption active sites can be replenished on the adsorbent after elution, it is expected to provide a novel and feasible solution for the efficient regeneration and sustainable application of metal-based adsorbents.

In this paper, the FeOOH/Al_2_O_3_ composites were synthesized via in situ growth of amorphous FeOOH within the pores of Al_2_O_3_ using a facile strategy. This strategy ingeniously leveraged the excellent pore structure of Al_2_O_3_ and the favorable affinity of amorphous FeOOH for phosphate, enabling the prepared composite to achieve a remarkably high adsorption capacity. Notably, a novel acid-impregnation regeneration method, based on the elution method, was developed. The method was repeatable, simple and easily implementable, which was well-suited to meet the demands of practical industrial applications. Specifically, the used FeOOH/Al_2_O_3_ adsorbent was eluted in HCl solution to remove the phosphate-adsorbed FeOOH. Subsequently, amorphous FeOOH was in situ regenerated into the pores of Al_2_O_3_ through a simple process. This method could also introduce different active components into the pores of Al_2_O_3_ according to actual needs, demonstrating its flexibility. Moreover, it offered a new approach to addressing the issue of active component loss during the elution and regeneration of other metal composites. Experimental verification showed that the FeOOH/Al_2_O_3_ maintained a relatively high adsorption capacity after four cycles of regeneration. Therefore, this study presented an adsorbent with high adsorption capacity and excellent regenerability for phosphate removal.

## 2. Results and Discussion

### 2.1. Characterization of Adsorbents

**Figure 1:**
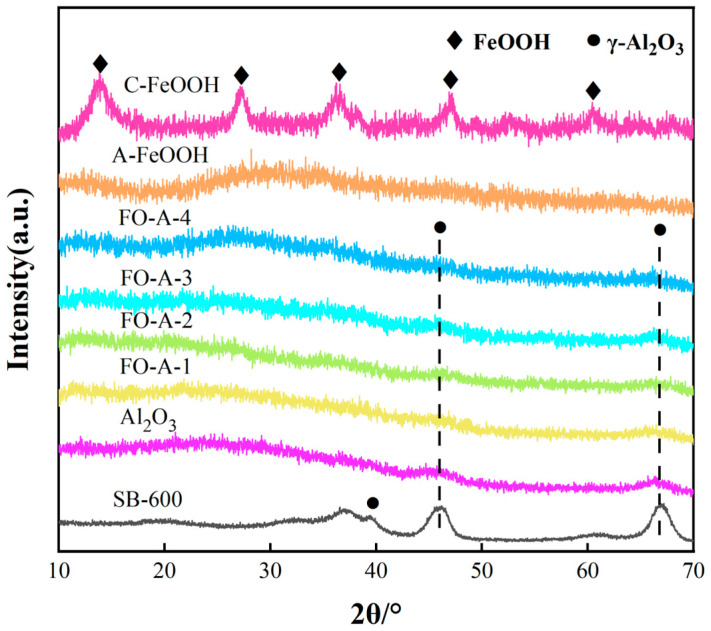
XRD patterns of FeOOH, FeOOH/Al_2_O_3_ (FO-A-x) and Al_2_O_3_ samples.

As presented in Figure 1, the characteristic diffraction peaks of γ-Al_2_O_3_ for Al_2_O_3_ were observed at 2θ of 45.8° and 66.8°, with no other diffraction peaks, indicating that the prepared sample was γ-Al_2_O_3_. SB-600 was the SB (Sasol boehmite) sample calcined at 600 °C for 2 h. Additionally, the characteristic peaks of γ-FeOOH for C-FeOOH were observed at 2θ of 14.15°, 27.07°, 36.39°, 46.87°, and 60.79°, confirming that the sample was γ-FeOOH. A-FeOOH was prepared by hydrolyzing a solution of FeCl_3_·6H_2_O at 100 °C for 5 h. During this process, the sample mass decreased significantly from the initial 2.7 g to 1.2 g, indicating a substantial mass loss. The XRD pattern of A-FeOOH showed no distinct diffraction peaks with a broadened and dispersed peak shape, suggesting an amorphous structure of A-FeOOH. It can be inferred that FeCl_3_·6H_2_O hydrolyzed at 100 °C to produce amorphous FeOOH, based on the corresponding mass loss during the hydrolysis process of A-FeOOH, as shown in Equation (1).

(1) $${\mathrm{FeCl}}_{3}\cdot 6H_{2}O\;\overset{100\;{}^{\circ}C}{\to }\;\mathrm{FeOOH}\cdot 1.67H_{2}O\;+\;3\mathrm{HCl}\;+\;2.33H_{2}O$$

XRD analysis of FO-A-x samples revealed no characteristic diffraction peaks of γ-FeOOH, confirming the in situ formation of amorphous FeOOH within the pores of Al_2_O_3_. Additionally, the primary crystal size of the C-FeOOH sample was calculated to be 3.8 nm using the Scherrer formula.

**Figure 2:**
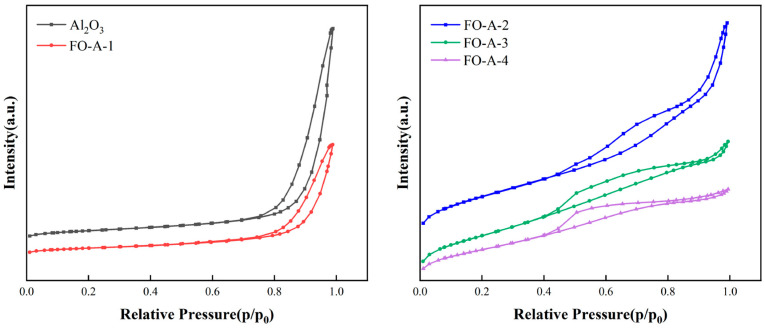
The N_2_ adsorption–desorption isotherms of samples.

**Figure 3:**
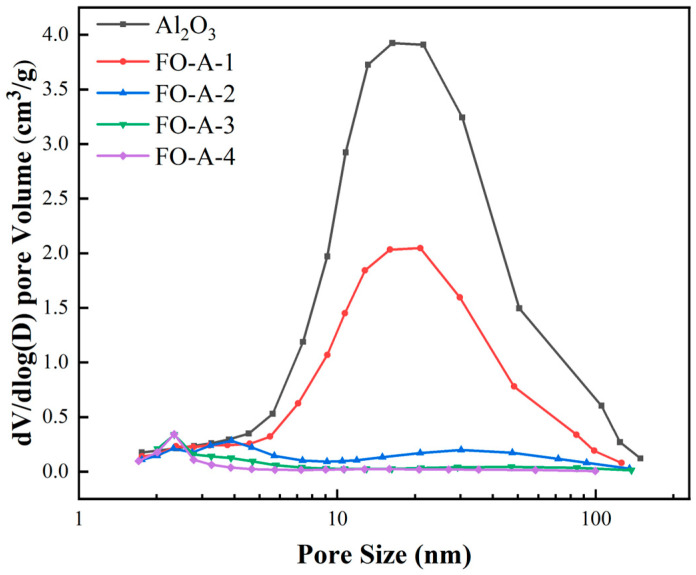
Pore size distribution of samples.

Figure 2 showed that all samples exhibited a Type IV adsorption–desorption isotherm, indicating the presence of mesopores. The hysteresis loops of Al_2_O_3_, FO-A-1, and FO-A-2 belonged to the H3-type, indicating cuneiform pores in these samples. However, the hysteresis loops of FO-A-3 and FO-A-4 belonged to the H4-type, suggesting narrow slit-like pores in these samples. Figure 3 depicts the differential pore size distribution curve of the sample, with the x-axis plotted on a logarithmic scale. Among the tested samples, Al_2_O_3_, FO-A-1, FO-A-2, and FO-A-3 displayed relatively broad pore size distributions, with most probable pore sizes of 16.3 nm, 20.8 nm, 3.9 nm, and 2.3 nm, respectively. However, FO-A-4 had a most probable pore size of 2.3 nm and exhibited a relatively concentrated pore size distribution. As shown in Table 1, BET analysis revealed that Al_2_O_3_ had a specific surface area of 493.7 m^2^/g and a pore volume of 3.62 cm^3^/g. As the molar ratio of Fe to Al increased from 0.10 to 0.54, the specific surface area of the sample gradually decreased from 307.9 m^2^/g to 182.7 m^2^/g, and the pore volume also declined from an initial value of 1.48 cm^3^/g to 0.20 cm^3^/g. The combination of XRD patterns indicated that amorphous FeOOH was grown in the pores of Al_2_O_3_ [33].

**Figure 4:**
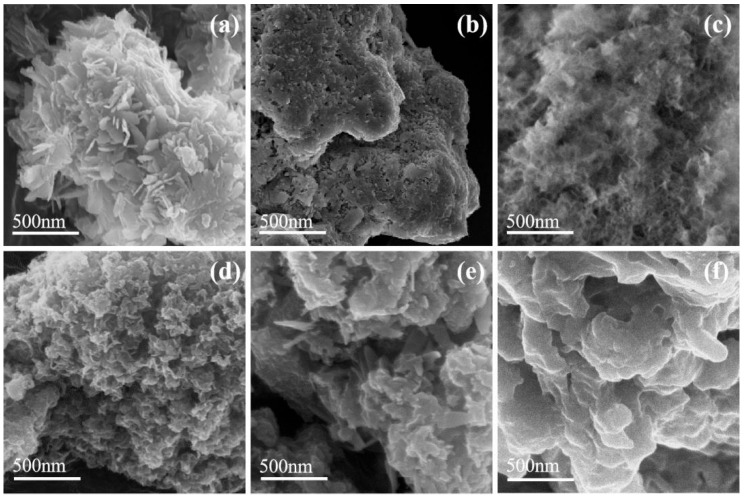
SEM images of samples ((**a**): C-FeOOH, (**b**): A-FeOOH, (**c**): Al_2_O_3_, (**d**): FO-A-1, (**e**): FO-A-3, (**f**): FO-A-4).

Figure 4 presents SEM images showing the morphology of the samples. C-FeOOH exhibited a multi-dimensional nanosheet morphology, as shown in Figure 4a. A-FeOOH displayed an aggregate structure, as shown in Figure 4b. As shown in Figure 4c, Al_2_O_3_ possessed a more intricate pore structure, with abundant voids retained between particles, which was consistent with the conclusion drawn from the BET analysis that Al_2_O_3_ had a larger pore volume. As depicted in Figure 4d–f, the pores of Al_2_O_3_ were obscured by densely packed FeOOH particles in FO-A-x samples, which were tightly interconnected with only minute gaps between them. As the FeOOH content increased, the covering layer became even thicker and denser. EDS characterization data for Fe and Al elements in FO-A-x samples were listed in Table 2. The actual molar ratios of Fe to Al in FO-A-1, FO-A-2, FO-A-3, and FO-A-4, which were obtained through EDS characterization were 0.08, 0.20, 0.45, and 0.56, respectively, which were relatively close to the theoretical values.

**Figure 5:**
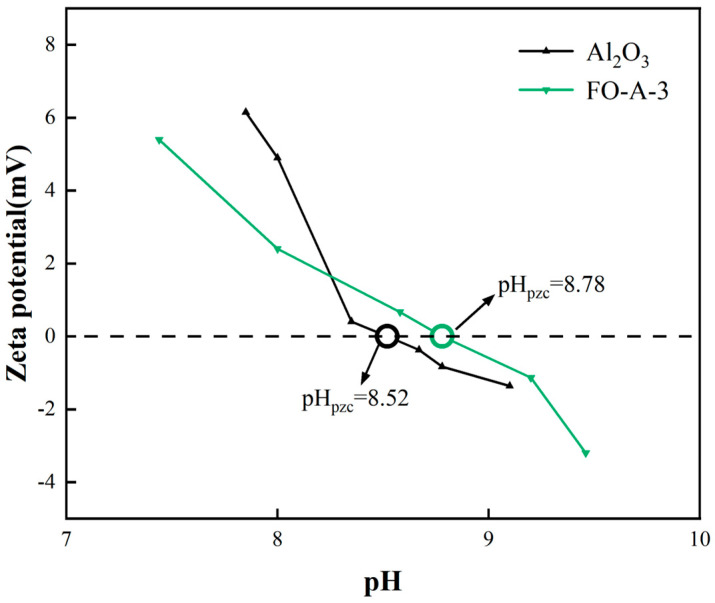
Isoelectric point of Al_2_O_3_ and FO-A-3.

Furthermore, due to the introduction of amorphous FeOOH, FO-A-3 exhibited an elevated isoelectric point (IEP) of 8.78, which was higher than that of Al_2_O_3_ (8.52), as shown in Figure 5. This increase in IEP indicated a greater abundance of surface hydroxyl groups, implying there were more active adsorption sites [34].

### 2.2. Adsorption Behavior

#### 2.2.1. Study on Adsorption Kinetics

**Figure 6:**
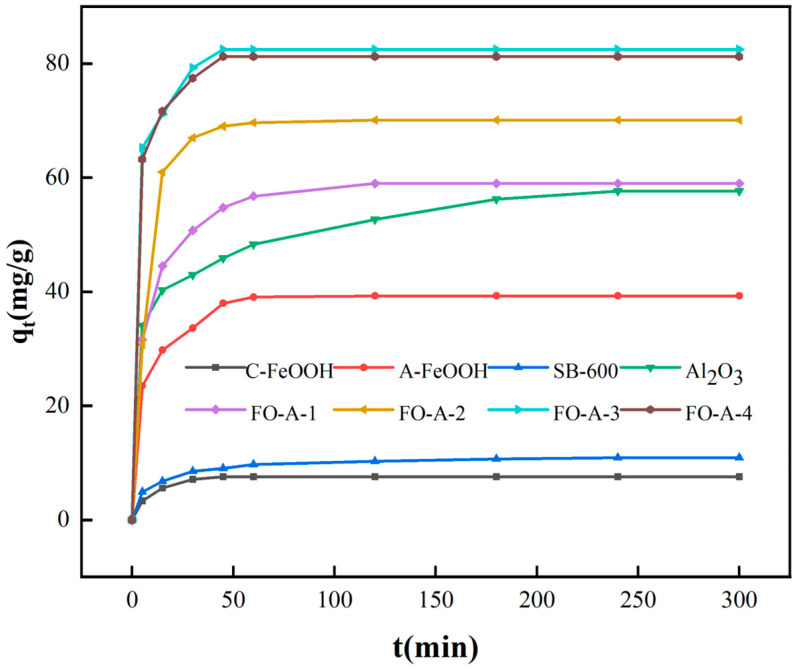
Adsorption capacity–time curve of samples for phosphate.

The adsorption capacity–time curve for phosphate is depicted in Figure 6. Notably, A-FeOOH exhibited an adsorption capacity of 39.27 mg/g, which was higher than that of C-FeOOH (7.56 mg/g). This is because the smaller primary crystal size of A-FeOOH led to more active adsorption sites, which were more favorable for adsorption. The synthesized Al_2_O_3_ in this study exhibited a higher adsorption capacity of phosphate compared to commercial Al_2_O_3_ (SB-600). Furthermore, the adsorption capacities of the other samples were FO-A-3 (82.47 mg/g) > FO-A-4 (81.24 mg/g) > FO-A-2 (70.11 mg/g) > FO-A-1 (58.98 mg/g) > Al_2_O_3_ (57.75 mg/g) > SB-600 (10.89 mg/g). Such performance is closely aligned with the increasing trend of the loading, mainly because the increased number of active adsorption sites provided by amorphous FeOOH. FO-A-3, prepared with the Fe to Al molar ratio of 0.47, exhibited the maximum adsorption capacity and a relatively fast adsorption rate. Further increasing the loading of amorphous FeOOH would trigger agglomeration, damaging the pore structure and decreasing the overall adsorption performance of the materials. As shown in Table 3, the kinetic data for C-FeOOH, A-FeOOH, Al_2_O_3_, FO-A-1, FO-A-2, and FO-A-3 conformed to the pseudo-second-order kinetic model, and the linear coefficient R^2^ approached 1, indicating that the adsorption process might exhibit chemisorption characteristics [35]. The experimental data of FO-A-4 aligned more closely with the pseudo-first-order model, which may be attributed to its relatively small specific surface area and pore volume.

#### 2.2.2. Adsorption Isotherm Models

**Figure 7:**
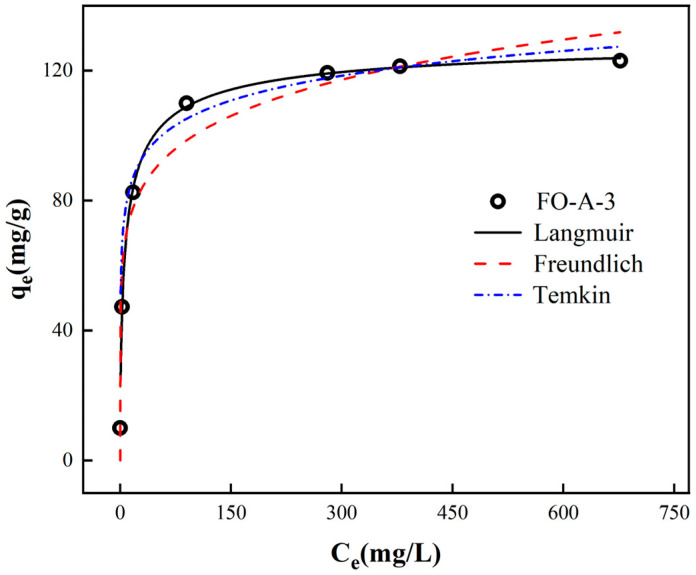
Adsorption isotherms results of the adsorption process of FO-A-3 to phosphate.

The corresponding fits to Langmuir, Freundlich, and Temkin isotherm models were depicted in Figure 7. As shown in Table 4, the Langmuir model exhibited a higher correlation coefficient, indicating that the adsorption of phosphate on FO-A-3 was a single-layer homogeneous adsorption process [36]. It was worth noting that the maximum adsorption capacity of FO-A-3 was 131.00 mg/g, which was higher than most of the values reported in the literature, as shown in Table 5.

#### 2.2.3. The Effect of Adsorbent Dosage

**Figure 8:**
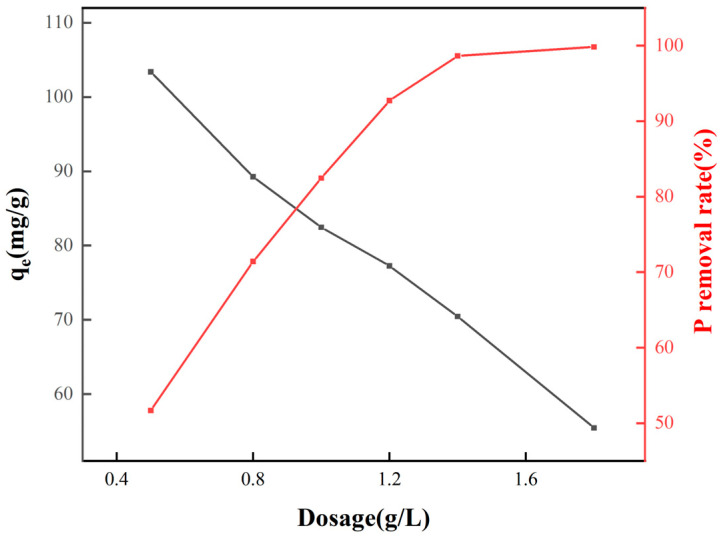
Effect of the dosage of FO-A-3 on the phosphate removal.

As shown in Figure 8, when the dosage of FO-A-3 increased from 0.5 g/L to 1.8 g/L, the equilibrium adsorption capacity of phosphate decreased from 103.37 mg/g to 55.47 mg/g, while the removal efficiency improved from 51.68% to 99.82%. The increase in the adsorbent dosage expanded the contact area between the adsorbent and phosphate, enabling more active sites to participate in the reaction, thereby enhancing the removal efficiency.

#### 2.2.4. The Effect of Initial pH

The initial pH of the solution plays a pivotal role in phosphate removal. On one hand, it influences the existing forms of phosphate across different pH ranges. Specifically, when the pH < 2.15, phosphate predominantly exists as electrically neutral H_3_PO_4_, which makes it impossible to be adsorbed through electrostatic attraction [1]. On the other hand, it can also affect the surface charge of adsorbents, thereby affecting the interaction between adsorbents and phosphate [13].

**Figure 9:**
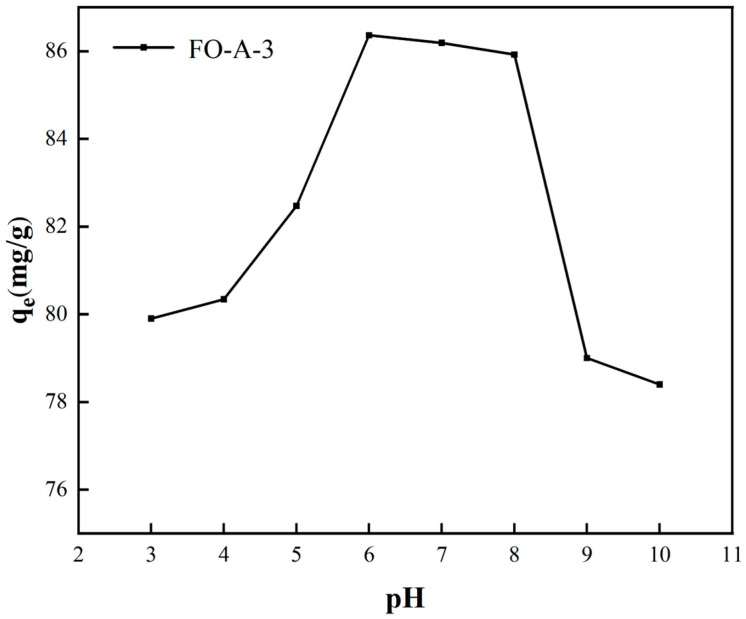
Effect of the initial solution of pH on the phosphate removal of FO-A-3.

As shown in Figure 9, when the pH of the solution decreased from 5.0 to 3.0, the adsorption capacity decreased from 82.47 mg/g to 79.90 mg/g. However, when the pH of the solution decreased from 10.0 to 6.0, the adsorption capacity increased from 78.40 mg/g to 86.36 mg/g. The exchange between hydroxyl groups on the surface of the adsorbent and phosphate, along with the formation of phosphate complexes, has been proven to be the primary process for phosphate removal [13]. The interaction between adsorbents and phosphate depended on the pH of the solution and the IEP of the adsorbents [31]. When the initial pH ranged from 6.0 to 8.0, the adsorption capacity slightly increased. This was because when the solution pH < IEP (the IEP of FO-A-3 was 8.78), the hydroxyl groups on the surface of FO-A-3 became protonated, making the surface positively charged and thereby resulting in strong electrostatic attractions [42]. However, when the solution pH < 6.0, a small portion of amorphous FeOOH would dissolve in the phosphate aqueous solution, exerting a negative impact on adsorption. Hence, FO-A-3 had a high adsorption capacity of 86.36 mg/g at pH = 6.0. Nevertheless, FO-A-3 exhibited a relatively high adsorption capacity (78.40–86.36 mg/g) at pH = 3.0–10.0.

### 2.3. Regeneration of FeOOH/Al_2_O_3_ Composites

**Figure 10:**
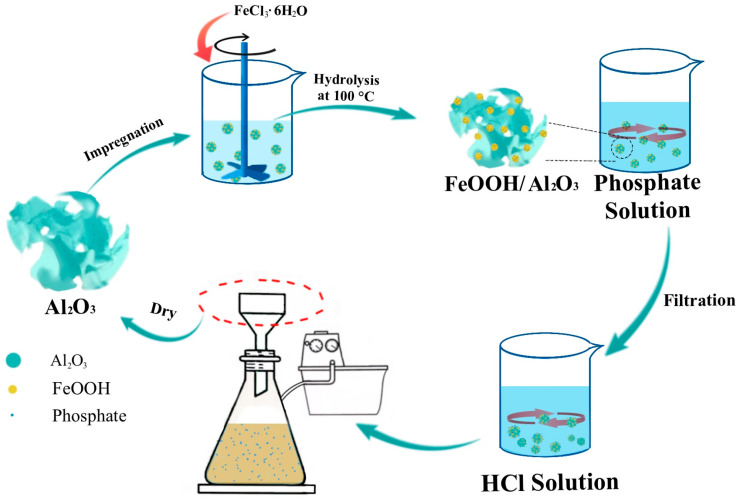
Schematic illustration of the regeneration process.

The regeneration process of FeOOH/Al_2_O_3_ using the acid-impregnation method was illustrated in Figure 10. The phosphate-adsorbed FeOOH/Al_2_O_3_ was eluted with an HCl solution (pH = 1.2), and the phosphate adsorbed on amorphous FeOOH was removed through the dissolution process of amorphous FeOOH in the HCl solution, yielding Al_2_O_3_. Then, FeCl_3_·6H_2_O solution was impregnated into the pores of Al_2_O_3_, and FeOOH/Al_2_O_3_ was reprepared via hydrolysis at 100 °C, thereby achieving adsorbent regeneration through a simple process.

**Figure 11:**
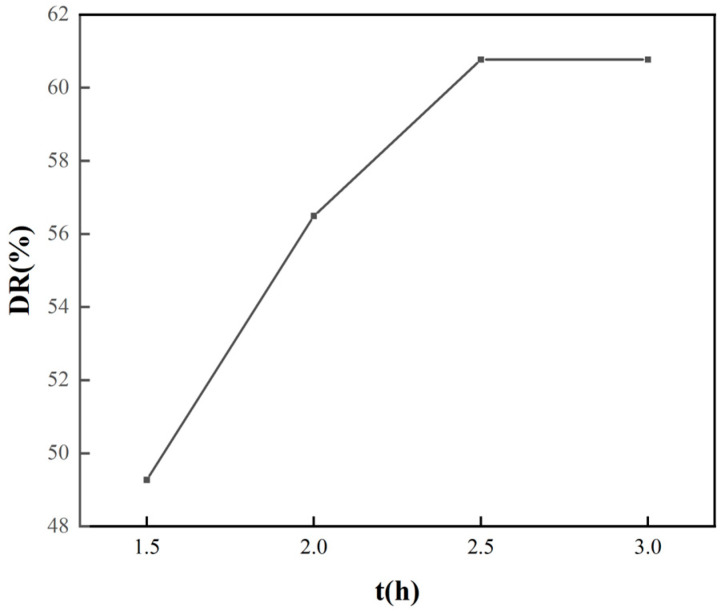
Desorption rate of FO-A-3 adsorbent at different elution times.

It was important to ensure a high desorption rate by controlling the elution time of the HCl solution. As shown in Figure 11, the desorption rates at an elution time of 1.5 h, 2 h, 2.5 h, and 3 h were 49.27%, 56.49%, 60.77%, and 60.77%, respectively, indicating that with the extension of elution time, more phosphate was desorbed, and equilibrium was basically reached at 2.5 h. To ensure the elution of more phosphate, a HCl solution with a pH of 1.2 was selected for an elution time of 2.5 h. Subsequently, the eluted sample was impregnated with FeCl_3_·6H_2_O solution after washing and drying.

**Figure 12:**
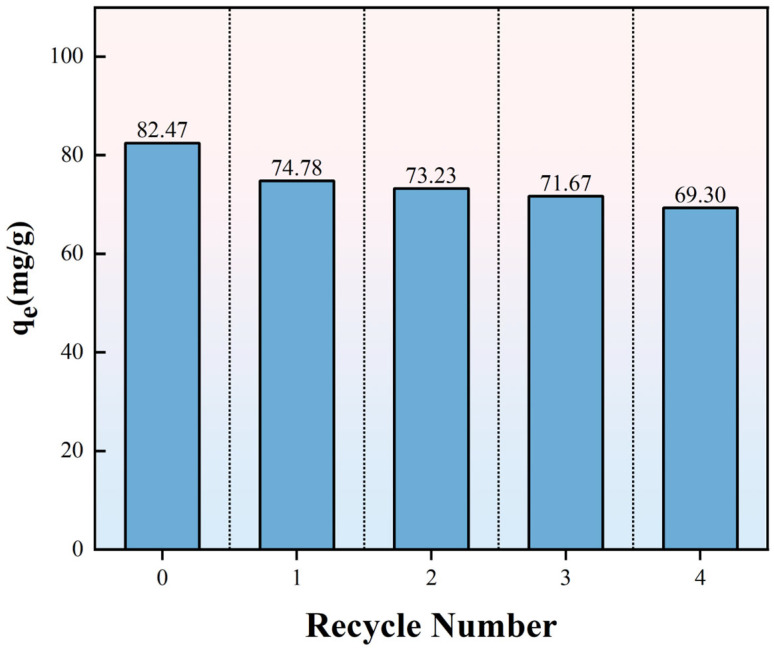
The relationship between the recycle number of FO-A-3 and the adsorption capacity.

As shown in Figure 12, the adsorption capacity of regenerated FO-A-3 was compared with that of the pristine FO-A-3. The adsorption capacity of the pristine FO-A-3 was 82.47 mg/g, while the adsorption capacities after four regeneration cycles were 74.78 mg/g, 73.23 mg/g, 71.67 mg/g, and 69.30 mg/g, respectively.

The ZJ-2 sample was obtained after two cycles of regeneration. As shown in Table 6, the specific surface area, pore volume, and most probable pore size of ZJ-2 were 169.7 m^2^/g, 0.18 cm^3^/g, and 2.4 nm, respectively. Compared with the adsorbent before regeneration, both its specific surface area and pore volume decreased, which resulted in a slight decrease in its adsorption capacity after regeneration. Overall, FO-A-3 maintained 84.03% of its original capacity, demonstrating excellent regeneration performance.

## 3. Materials and Methods

### 3.1. Materials

Aluminum sulfate octadecahydrate (Al_2_(SO_4_)_3_·18H_2_O), aluminum hydroxide (Al(OH)_3_), sodium hydroxide (NaOH), and potassium dihydrogen phosphate (KH_2_PO_4_) were obtained from Xilong Scientific Company Limited (Shantou, China). Ferric chloride hexahydrate (FeCl_3_·6H_2_O), ammonia solution (NH_3_·H_2_O), and hydrochloric acid (HCl) were obtained from Tianjin Fuchen Chemical Reagents Factory Company Limited (Tianjin, China). All the chemicals were of analytical reagent grade. Sasol boehmite (SB) was sourced from Sasol Germany GmbH (Hamburg Germany).

### 3.2. Synthesis

#### 3.2.1. Preparation of Al_2_O_3_

Al_2_O_3_ was prepared by calcining a boehmite sample at 600 °C for 2 h. The preparation method of boehmite followed the procedure described in Reference [43].

#### 3.2.2. Preparation of SB-600

Commercial Al_2_O_3_, denoted as SB-600, was prepared by calcining SB (Sasol boehmite) at 600 °C for 2 h.

#### 3.2.3. Preparation of Amorphous FeOOH and Crystalline FeOOH

Amorphous FeOOH, denoted as A-FeOOH, was prepared as follows: First, 0.009989 mol (2.70 g) of FeCl_3_·6H_2_O was added to 1.5 mL H_2_O in a beaker and stirred at 400 rpm to complete dissolution of the solid materials. Then, the solution was hydrolyzed in an oven at 100 °C for 5 h.

Crystalline FeOOH, denoted as C-FeOOH, was prepared as follows: First, 1.2 g of A-FeOOH was added to a beaker containing 45.0 mL of deionized water and thoroughly mixed at a stirring speed of 400 rpm. Subsequently, a 0.0609 mol/L ammonia solution was added dropwise to the above solution under magnetic stirring at 400 rpm to adjust the pH value to 10.9. Afterwards, the mixture was transferred into a Teflon-lined stainless steel autoclave and stirred at 20 rpm for 2 h in a homogeneous reactor at 95 °C for dynamic crystallization. Finally, the product was obtained by washing with water and ethanol and drying at 80 °C for 2.5 h.

#### 3.2.4. Preparation of FeOOH/Al_2_O_3_ Composites

0.003922 mol (1.06 g), 0.009020 mol (2.44 g), 0.01843 mol (4.98 g), and 0.02235 mol (6.04 g) of FeCl_3_·6H_2_O were added separately to 1.5 mL deionized water and stirred at 400 rpm until dissolved to prepare a series of impregnation solutions. The impregnation solutions were then sequentially impregnated into 0.01961 mol (2.00 g) of Al_2_O_3_ (prepared as described in Section 3.2.1) and then hydrolyzed in an oven at 100 °Cfor 5 h to obtain FeOOH/Al_2_O_3_ composites, which were recorded as FO-A-x. The prepared composites exhibited molar ratios of Fe to Al of 0.10, 0.23, 0.47, and 0.57 in sequence, and these composites were named FO-A-1, FO-A-2, FO-A-3, and FO-A-4, respectively.

### 3.3. Analytical Procedures

The phase composition of the material was analyzed using a D8 Advanced Bruker diffractometer (Bruker, Karlsruhe, Germany) with Cu-Kα radiation (λ = 0.15406 nm) applied in the 2θ range of 10° to 70°. The primary crystal size could be calculated using the Debye-Scherrer equation (Equation (2)).

(2) $$D_{hkl}=\frac{0.89\lambda }{\sqrt{\left(B_{m}^{2}{-B}_{s}^{2}\right)}cos\theta }$$

where D_hkl_ is the average size corresponding to the (hkl) plane, λ is the wavelength of X-rays, B_m_ is the full width at half maximum (FWHM) of the sample at the diffraction peak, B_s_ is the full width at half maximum (FWHM) of high-purity quartz, and θ is the Bragg angle.

The specific surface area and pore structure characteristics of the materials were determined using the nitrogen adsorption–desorption method combined with the Brunauer-Emmett-Teller (BET) analysis on a TriStar II 3020 instrument (Micromeritic, Norcross, GA, USA). The morphological features of the materials were examined using a SUPRA 55 scanning electron microscope (Carl Zeiss AG, Karlsruhe, Germany). The samples were dispersed in a 0.01 M KNO_3_ solution, after which the pH was fine-tuned using 0.01 M HNO_3_ and 0.01 M KOH solutions. Subsequently, zeta potential measurements at the corresponding pH values were performed using a ZEN3600 Malvern instrument (Malvern, Marvin City, UK). The aqueous solution containing residual phosphate was tested by ammonium molybdate spectrophotometry using a Shimadzu UV-2600 ultraviolet-visible spectrophotometer (Shimadzu, Kyoto, Japan).

### 3.4. Adsorption and Desorption Studies

All adsorption experiments were conducted on a magnetic stirrer at room temperature and a stirring speed of 400 rpm. The aqueous solution containing residual phosphate was sampled through filtration. The equilibrium adsorption capacity (q_e_, mg/g) and the removal efficiency (Re, %) of phosphate were calculated by the following equations (Equations (3) and (4)).

(3) $$q_{e}=\frac{\left(C_{0}-C_{e}\right)\times V}{m}$$

(4) $$Re=\frac{\left(C_{0}-C_{e}\right)\times 100\%}{C_{0}}$$

where C_0_ (mg/L) is the initial concentration of phosphate, C_e_ (mg/L) is the equilibrium concentration of phosphate, V (L) is the total volume of the solution, and m (g) is the mass of the adsorbent.

#### 3.4.1. Adsorption Kinetics and Isotherm

To evaluate the adsorption kinetics, 50 mg of adsorbent was added to 50 mL of phosphate aqueous solution with a concentration of 100 mg/L (calculated by P). In the experiments, the time intervals for the adsorption process were set at 5, 15, 30, 45, 60, 120, 180, 240, and 300 min, respectively. The experimentally obtained data were fitted to the pseudo-first-order (Equation (5)), pseudo-second-order (Equation (6)) kinetic models.

(5) $$\mathrm{lg}\left(q_{e}-q_{t}\right)=lgq_{e}-\frac{k_{1}}{2.303}t$$

(6) $$\frac{t}{q_{t}}=\frac{1}{k_{2}{q_{e}}^{2}}+\frac{1}{q_{e}}t$$

where q_t_ (mg/g) represents the phosphate adsorption capacity at time t (min), k_1_ (min^−1^) and k_2_ (g/mg·min) are the kinetic constants of the pseudo-first-order and pseudo-second-order models, respectively.

The determination of the adsorption isotherm involved contacting 50 mg of adsorbent with 50 mL of phosphate aqueous solution at concentrations of 10, 50, 100, 200, 400, 500, and 800 mg/L until equilibrium was achieved. Subsequently, the experimental data were fitted to Langmuir (Equation (7)), Freundlich (Equation (8)), and Temkin (Equation (9)) isotherm models.

(7) $$q_{e}=\frac{q_{m}\times K_{L}\times C_{e}}{1+K_{L}\times C_{e}}$$

(8) $$q_{e}={K_{F}\times C}_{e}^{1/n}$$

(9) $$q_{e}=\frac{RT}{b_{T}}lnK_{T}+\frac{RT}{b_{T}}lnC_{e}$$

where q_m_ (mg/g) is the theoretical maximum adsorption capacity of the adsorbent, K_L_ and K_F_ are the constants of the Langmuir and the Freundlich models, respectively, b_T_ (J/mol) and K_T_ (L/g) are the constants of the Temkin isotherm.

#### 3.4.2. Effect of Adsorbent Dosage and the Initial pH

The impact of varying adsorbent dosages and initial pH on phosphate removal performance was investigated using a 100 mg/L phosphate aqueous solution at room temperature. Specifically, 25, 40, 50, 60, 70, and 90 mg of the adsorbent were separately added to 50 mL of phosphate aqueous solution. Furthermore, to explore the effect of pH, 50 mg of adsorbent was added to 50 mL of phosphate aqueous solution, and the initial pH was adjusted to a range of 3.0 to 10.0 using 0.10 M HCl solution and 0.10 M NaOH solution.

#### 3.4.3. Regeneration Performance

To achieve good regeneration of FeOOH/Al_2_O_3_ adsorbent, for FO-A-3, 1 g of the phosphate-adsorbed FO-A-3 adsorbent was added to 1 L of HCl solution (pH = 1.2) and continuously eluted in a magnetic stirrer at 400 rpm for 2.5 h. After the elution, the precipitate was collected and washed with deionized water until the filtrate reached neutrality. Subsequently, it was washed twice with ethanol and dried to obtain Al_2_O_3_. Next, 0.01843 mol (4.98 g) of FeCl_3_·6H_2_O and 1.5 mL of deionized water were stirred at 400 rpm to prepare an impregnation solution matching those used for synthesizing FO-A-3 adsorbent. Then, this solution was impregnated into the pores of 2 g of the obtained Al_2_O_3_. Finally, the mixture was placed in an oven at 100 °C for 5 h to obtain the regenerated adsorbent for the next adsorption experiment. The sample obtained after two cycles of regeneration was named ZJ-2. The desorption capacity (q_d_) and the desorption rate (DR) of the adsorbent were calculated by the following equations (Equations (10) and (11)).

(10) $$q_{d}=\frac{V^{\prime }\times c_{e}^{\prime }}{m\prime }$$

(11) $$DR=\frac{q_{d}\times 100\%}{q_{e}}$$

where V′ (L) is the volume of the elution solution, m′ (g) is the mass of the phosphate-adsorbed adsorbent, $c_{e}^{\prime }$ (mg/L) is the concentration of phosphate in the elution solution.

## 4. Conclusions

In this study, an FeOOH/Al_2_O_3_ composite was successfully synthesized by in situ generating amorphous FeOOH within the pores of Al_2_O_3_, and the reaction process for the formation of amorphous FeOOH was deduced. During the experiments, we comparatively investigated the phosphate adsorption performance of self-made versus commercial Al_2_O_3_, crystalline versus amorphous FeOOH, as well as a series of FeOOH/Al_2_O_3_ samples. The results revealed that the self-made Al_2_O_3_ exhibited a higher adsorption capacity than the commercial one, and the amorphous FeOOH demonstrated a greater adsorption capacity compared to crystalline FeOOH. FO-A-3, one of the FeOOH/Al_2_O_3_ samples prepared with an Fe/Al molar ratio of 0.47, exhibited excellent adsorption capacity and a relatively fast adsorption rate, surpassing those of Al_2_O_3_ and amorphous FeOOH alone. The phosphate adsorption process using FO-A-3 conformed to the pseudo-second-order kinetic model and the Langmuir isotherm model, with a maximum adsorption capacity of 131.00 mg/g. Additionally, we examined the effects of factors such as the initial pH of the solution and the dosage of FO-A-3 on phosphate adsorption using FO-A-3 adsorbent. This study innovatively developed a regeneration method that was simple and highly flexible. Experiments demonstrated that after four regeneration cycles, the adsorption capacity of FO-A-3 could still maintain 84.03% of its initial capacity. The research results indicated that FO-A-3 exhibited significant potential as an adsorbent for effectively removing phosphate from wastewater.

## Figures and Tables

**Table 1:** Data sheet of BET analysis.

Adsorbent	BET Surface Area (m^2^/g)	BET Pore Volume (cm^3^/g)	Most Probable Pore Size (nm)
Al_2_O_3_	493.7	3.62	16.3
FO-A-1	307.9	1.48	20.8
FO-A-2	264.6	0.45	3.9
FO-A-3	235.0	0.28	2.3
FO-A-4	182.7	0.20	2.3

**Table 2:** Elemental composition of Fe and Al in FO-A-3 sample via EDS.

Adsorbent	Fe Atomic Fraction (%)	Al Atomic Fraction (%)	nFe^3+^/nAl^3+^ EDS
FO-A-1	2.41	25.22	0.08
FO-A-2	1.15	20.77	0.20
FO-A-3	2.33	20.67	0.45
FO-A-4	2.25	19.98	0.56

**Table 3:** The kinetic fitting data table of the adsorption process of phosphate by samples.

Adsorbent	Q_e,exp_ (mg/g)	Pseudo-First-Order			Pseudo-Second-Order		
		q_e_ (mg/g)	k_1_ (min^−1^)	R^2^	q_e_ (mg/g)	k_2_ (g/(mg∙min))	R^2^
C-FeOOH	7.56	8.03	0.1641	0.9047	7.80	0.03197	0.9989
A-FeOOH	39.27	31.78	0.07623	0.9471	40.32	0.006276	0.9995
Al_2_O_3_	57.75	57.64	0.01520	0.9835	59.52	0.001610	0.9987
FO-A-1	59.98	31.30	0.04445	0.9950	60.98	0.003244	0.9998
FO-A-2	70.11	39.93	0.07807	0.9743	71.94	0004342	0.9992
FO-A-3	82.47	26.80	0.06840	0.9725	85.47	0.005265	0.9993
FO-A-4	81.24	24.51	0.06241	0.9999	84.03	0.005510	0.9995

**Table 4:** The isotherm models fitting data table of adsorption to phosphate.

	Langmuir			Freundlich			Temkin		
Adsorbent	q_m,cal_ (mg/g)	K_L_ (L/mg)	R^2^	K_F_	1/n	R^2^	b_T_ (J/mol)	K_T_ (L/mg)	R^2^
FO-A-3	131.00	0.2964	0.9987	51.4151	0.1445	0.9488	206.3171	157.03	0.9238

**Table 5:** Comparison of the maximum adsorption capacity of phosphate by different adsorbents.

Adsorbent	Adsorption Capacity mg/g	References
P500K1.5–500	58.72	[36]
BC-G	22.14	[37]
4:1 Mg/Al-LDHs	81.83	[38]
Amorphous FeOOH	115.61	[24]
MALZ	80.80	[32]
Fe_3_O_4_@MIL-100(Fe)@Mg-Al LDH	54.43	[39]
La-Zr@SA/NIPAM	105.26	[40]
Mg/Al-LDH@HC-HCl	143.03	[41]
FeOOH/BF	39.08	[26]
FeOOH@MS	115.50	[30]
Activated aluminum oxide	20.88	[12]
FO-A-3	131.00	This study

**Table 6:** Data sheet of BET for the sample after two regeneration cycles.

Adsorbent	BET Surface Area (m^2^/g)	BET Pore Volume (cm^3^/g)	Most Probable Pore Size (nm)
ZJ-2	169.7	0.18	2.4

## Data Availability

The original contributions presented in this study are included in the article. Further inquiries can be directed to the corresponding author.
